# Salinity shapes microbial diversity and community structure in surface sediments of the Qinghai-Tibetan Lakes

**DOI:** 10.1038/srep25078

**Published:** 2016-04-26

**Authors:** Jian Yang, Li’an Ma, Hongchen Jiang, Geng Wu, Hailiang Dong

**Affiliations:** 1 State Key Laboratory of Biogeology and Environmental Geology, China University of Geosciences, Wuhan, 430074, China; 2 College of Life Science, Yangtze University, Jingzhou, Hubei 434025, China; 3 Department of Geology and Environmental Earth Science, Miami University, Oxford, OH45056, USA

## Abstract

Investigating microbial response to environmental variables is of great importance for understanding of microbial acclimatization and evolution in natural environments. However, little is known about how microbial communities responded to environmental factors (e.g. salinity, geographic distance) in lake surface sediments of the Qinghai-Tibetan Plateau (QTP). In this study, microbial diversity and community structure in the surface sediments of nine lakes on the QTP were investigated by using the Illumina Miseq sequencing technique and the resulting microbial data were statistically analyzed in combination with environmental variables. The results showed total microbial community of the studied lakes was significantly correlated (r = 0.631, *P* < 0.001) with lake salinity instead of geographic distance. This suggests that lake salinity is more important than geographic distance in shaping the microbial diversity and community structure in the studied samples. In addition, the abundant and rare taxa (OTUs with relative abundance higher than 1% and lower than 0.01% within one sample, respectively) were significantly (*P* < 0.05) correlated (r = 0.427 and 0.783, respectively) with salinity, suggesting rare taxa might be more sensitive to salinity than their abundant counterparts, thus cautions should be taken in future when evaluating microbial response (abundant vs. rare sub-communities) to environmental conditions.

Inland waters (e.g. lakes, rivers, wetlands, reservoirs) are of great importance for understanding the impact of human activity and climate change on ecosystem structures and functions, although they occupy a small fraction (~1%) of the earth’s surface^1^. Microbes, possessing plenty of genetic diversity, are a fundamental component of aquatic ecosystems and play essential roles in global biogeochemical cycles^2^. Therefore, studying microbial diversity in inland aquatic ecosystems provides insight into the biogeochemical processes and ecological mechanisms that maintain ecosystem functions^3^.

Saline lakes account for approximately a half of total inland aquatic ecosystems^4^. In saline lakes, microbial metabolisms are extremely active and involve the geochemical cycles of many life-essential elements such as carbon, nitrogen and sulfur^5^. Microbial activities in saline lakes are mainly controlled by salinity due to energetic constraints^6^, and thus salinity should be a dominant factor in shaping microbial diversity and community composition^7^. Many previous studies have shown that salinity influences microbial community composition (MCC) in lake waters^8^,^9^,^10^. However, the effect of salinity on MCC in lake surface sediments remains poorly understood^11^, possibly because microbes in lake sediment may not be so sensitive to salinity change as that in lake water.

Lake surface sediments host diverse microbial communities, which are important to maintaining the benthic food web structure as well as function (e.g. biogenic elemental cycling)^12^,^13^. Microbes in lake surface sediments undergo similar osmotic pressure to that in lake waters. So it is possible that salinity also affects the MCC of lake surface sediments. However, no clear salinity effect was ever observed on MCC of lake surface sediments up to date^14^,^15^.

In addition, microbial community in natural environments could be classified into abundant and rare taxa with respect to their contribution to biomass and biodiversity^16^. Abundant taxa contribute major biomass but minor biodiversity to the ecosystem, whereas rare taxa contribute minor biomass but major biodiversity^16^. The distribution of abundant and rare taxa in lake waters has been evaluated although some debates exist. For example, a recent study indicated that geographic distance dominated distribution of both abundant and rare microbial communities in lake waters (freshwater) with the latter exhibiting a stronger correlation with geographic distance than the former^3^. In contrast, another study suggested that salinity affected distribution of abundant and rare microbes in waters of coastal lakes (salinity range: 0–100 g/L)^17^. However, it is poorly known that how abundant and rare MCCs respond to geographic distance in lakes with a larger salinity range (e.g. from freshwater to salt-saturation). So it is necessary to reevaluate the effects of geographic distance and salinity on abundant and rare taxa in lakes with a large salinity range from freshwater to salt-saturation. Furthermore, up to date no studies have reported on the distribution patterns of abundant and rare taxa in lake surface sediments. Thus almost nothing is known about how geographic distance and/or salinity influence the distribution patterns of abundant and rare microbial communities in surface sediments of lakes with a large salinity range (up to salt saturation).

The Qinghai-Tibetan Plateau (QTP) is the largest (2 × 10^6^ km^2^) and highest (average ∼4500 meters above sea level) plateau on the Earth. It contains thousands of saline/hypersaline lakes, which possess a broad range of environmental gradients such as salinity (from 0.1 to 426.3 g L^−1^) and pH (5.4–10.2)^18^. Furthermore, the QTP lakes represent the most pristine natural environments and thus receive minimal human influence, which avails to study the effects of natural environmental variables (e.g. geographic distance, salinity) on microbial communities (abundant vs. rare taxa). The purposes of this study are 1) to investigate the salinity effect on MCC (including total, abundant and rare communities) in surface sediments of the QTP lakes, and 2) to discern which factor (geographic distance vs.salinity) significantly influences the distribution of total, abundant and rare MCCs, respectively.

## Results

### Geochemistry of the studied lakes

The salinity of the sampled lakes ranged from 0.6–324.8 g/L (Table 1). EHL (Erhai Lake) and KLKL (Keluke Lake) are freshwater (salinity <1 g/L); QHL(Qinghai Lake), GHL1 (Gahai Lake1) and TSL (Tuosu Lake) are saline (1 g/L < salinity < 35 g/L); and GHL2 (Gahai Lake2), XCDL (Xiaochaidan Lake), DBXL (Dabuxun Lake) and CKL(Chaka Lake) are hypersaline (salinity >35 g/L). The pH of these lakes was 7.0–9.4 (Table 1), negatively correlated with salinity (r = −0.916, *P* = 0.0004). Sediment TOC contents were 0.3–5.6% (Table 1).

### Microbial diversity of the studied lakes

A total of 702,216 high-quality sequences with 61,798–107,066 sequences (mean = 78,024) and 702–3650 OTUs (mean = 2317) for each sample (Table S1 in the supplementary material) were obtained. The diversity indices, including Shannon (3.3–6.4), phylogenetic distance of a whole tree (14.6–106.8), and Chao 1 (1012.1–4499) were shown in the supplementary material (in Table S1). All the calculated diversity indices in this study decreased with increasing salinity of the studied lakes (in Table S1). The dominant phyla (average relative abundance >1%) in the studied sediments were *Euryarchaeota*, *Acidobacteria*, *Actinobacteria*, *Bacteroidetes*, *Chloroflexi*, *Cyanobacteria*, *Firmicutes*, *Gemmatimonadetes*, *Planctomycete*, *Proteobacteria*, *Thermi*, and *Verrucomicrobia* (Fig. S1 in the supplementary material). *Proteobacteria* is the most abundant phylum (more than 60% of total sequence reads). The relative abundances of *Gammaproteobacteria* (r = 0.872) and *Clostridia* (r = 0.713) were significantly (*P* < 0.05) correlated with salinity.

Among the retrieved OTUs, a total of 8 - 19 OTUs were classified as abundant OTUs. These abundant OTUs accounted for 0.3–2.9% of total OTUs and represented 20.4–78.8% relative abundance of sequence reads in the studied samples (Supplementary Table S2). In contrast, a total of 426 −1997 rare OTUs were identified and they accounted for 59.8–78.8% of total OTUs and 1.5–8.7% relative abundance of sequence reads in the studied samples (Supplementary Table S2). Most abundant and rare OTUs belonged to *Proteobacteria* and *Bacteroidetes*: the abundant *Proteobacteria* and *Bacteroidetes* OTUs accounted for 18.2–60.9% and 0.0–8.3% of total sequence reads in the studied samples, respectively; while their rare counterparts accounted for 0.8–3.5% and 0.1–0.9% of total sequences, respectively (Supplementary Table S3 and S4).

**Figure 1:**
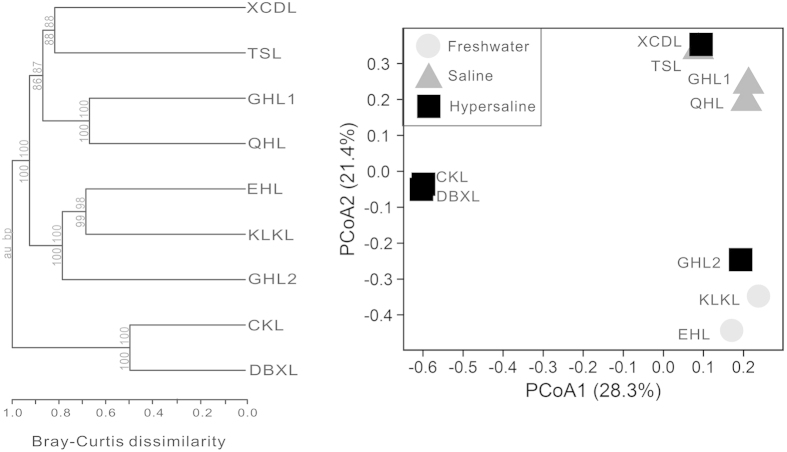
Clustering and principal coordinates analysis of total MCC among the studied samples based on Bray-Curtis dissimilarity.

The studied lake sediments with similar salinity showed similar total MCC patterns. For example, cluster analysis revealed that the two lakes (DBXL and CKL) with highest salinity (salinity >300 g/L) were grouped into one cluster which is distinct from the other lakes. Freshwater lakes (EHL and KLKL) were grouped into one cluster, and other mid-salinity lakes were grouped into two clusters (except for GHL2) (Fig. 1). The freshwater lake sediments (KLKL and EHL) were dominated by sequences affiliated with *Betaproteobacteria*, *Deltaproteobacteria*, *Gammaproteobacteria, Bacteroidia* and *Anaerolineae*; The saline lake sediments (QHL, TSL and GHL1) were dominated by sequences affiliated with *Alphaproteobacteria*, *Betaproteobacteria*, *Deltaproteobacteria*, *Gammaproteobacteria* and *Verrucomicrobiae*; while hypersaline lake sediments GHL2 and XCDL were dominated by *Alphaproteobacteria*, *Betaproteobacteria*, *Deltaproteobacteria*, *Gammaproteobacteria, Bacteroidia*, and the two almost salt-saturated DBXL and CKL samples were dominated by *Gammaproteobacteria* and *Clostridia* sequences (Table S5 in the supplementary material). Furthermore, the abundant and rare sub-communities in the studied lake surface sediments showed similar community composition-salinity patterns to total microbial community (Supplementary Fig. S2).

### Statistical analyses

**Figure 2:**
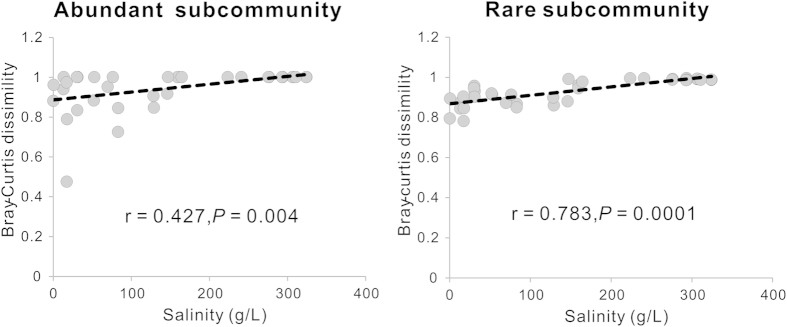
Pearson correlation between abundant/rare subcommunity dissimilarity and lake salinity.

Statistical analyses further corroborated the influence of salinity on the MCC in the studied lake sediments. Bray-Curtis dissimilarity of total microbial community was significantly correlated (r = 0.631, *P* < 0.001) with lake salinity, whereas no significant correlation was found between community dissimilarity and geographic distance (data not shown). Similarly, Mantel test showed that total MCC of the lake sediments was strongly correlated (*P* < 0.05) with salinity (r = 0.631) rather than geographic distance (Supplementary Table S6). Furthermore, Bray-Curtis dissimilarity of abundant and rare microbial communities were significantly correlated (r = 0.427 and r = 0.783, respectively) with lake salinity (Fig. 2) instead of geographic distance (data not shown). Likewise, Mantel test indicated that the abundant and rare MCCs were significantly correlated to salinity (r = 0.427 and r = 0.783) rather than geographic distance (Supplementary Table S6). In addition, Mantel tests also showed that the total and rare MCCs were significantly correlated with pH (r = 0.402 and r = 0.574) (Supplementary Table S6).

## Discussion

Salinity was an important factor influencing the microbial diversity and community structures in the surface sediments of the studied lakes. This finding was inconsistent with a recent study, in which geographic distance, rather than salinity, was shown to mostly influence sediment MCC of the QTP lakes^14^. Such inconsistency could be ascribed to different distances (4–467 km vs. 4–1670 km)^14^ among the studied lakes in the present and that studies^14^, respectively. The impact of spatial factors on microbial distribution pattern is likely scale dependent^19^. For example, at small spatial scales (<500 km), local environmental variables were frequently reported as the major factors influencing MCC; while at scales of ten to thousands of kilometers, spatial factor played an important role in shaping microbial community variation^20^. So it is reasonable to observe the salinity effect on MCC in the studied lakes (with small spatial distance). Such salinity impacts on MCC in lakes were also observed in previous studies^9^,^21^,^22^,^23^. Additionally, Mantel test showed that total MCC was also significantly correlated with pH of the studied lakes in this study, but the mantel correlation coefficient was lower than that for salinity (r = 0.402 vs. r = 0.631, Supplementary Table S6). Most known bacterial strains can grow well under pH 7.0–9.4 instead of a wide range of salinity (freshwater to salt saturation)^24^. Therefore, it is reasonable to conclude that salinity is more important than pH for influencing the MCC distribution in surface sediments of the studied QTP lakes.

In addition to salinity and pH, some unmeasured parameters may also contribute to shape the microbial community structures in the studied lakes. Two hypersaline lakes (GHL2 and XCDL) were clustered together with freshwater (EHL and KLKL) and saline (TSL) lakes (Fig. 1), suggesting that some similar microbial OTUs might be shared between freshwater and saline lakes. Our previous studies also showed that some microbial OTUs could be present in both freshwater and hypersaline lakes although their abundances varied with salinity^22^,^25^,^26^. The reason for such phenomena is that some microorganisms are capable of tolerating a large salinity range. For example, *Halomonas boliviensis*-like (16S rRNA gene identity >99%) isolates as well as 16S rRNA gene sequence reads were retrieved from XCDL, GHL2, EHL, KLKL and TSL (data not shown). *Halomonas boliviensis* was isolated from a hypersaline lake and was able to tolerate a salinity range of 0–25%^27^. However, the influencing factors remain uncertain for the distribution of such microbes with large salinity tolerance, which awaits further investigation.

It is remarkable that salinity rather than geographic distance significantly affected the structures of both abundant and rare microbial communities in the present study. This point was not consistent with a recent study of freshwater lakes, which indicated that the distribution of both abundant and rare bacteria in lake waters were significantly correlated with geographic distance^3^. The possible reason for such inconsistency is that a large salinity range (freshwater to almost salt saturation) in this study may strongly constrain the dispersal of abundant and rare species due to energy limitation^6^.

It is also notable that the rare subcommunities in the present study exhibit more evident response (as evidenced by the larger correlation coefficients) to salinity than their abundant counterparts. This suggested that rare taxa may exhibit more restricted distribution along a salinity range than their abundant counterparts. Abundant taxa can utilize a wide spectrum of resources and thus^28^ have low probability of extinction and high probability of dispersal^3^. In addition, rare taxa may occupy less suitable micro-niches in sediments and are thus more easily affected by environmental conditions (including salinity) than their abundant counterparts. Therefore, rare taxa might respond more sensitively to salinity and other environmental conditions than abundant taxa. Taken together, cautions should be taken when evaluating microbial response (response signals from abundant vs. rare taxa) to environmental variables.

In summary, our data show that salinity is the most important factor shaping microbial diversity and structure regardless of abundant or rare sub-communities in surface sediments of the Qinghai-Tibetan Lakes. Rare taxa are more sensitive to salinity (possibly including other environmental conditions) than their abundant counterparts.

## Methods and Materials

### Sampling and geochemistry measurements

In summer 2010, nearshore sediments were collected from nine lakes on the northern Tibetan Plateau with a grab-bucket collection sampler (Table 1). The water depth of sampling sites was approximately 1.5 meter (Table 1). Specifically, surface lake sediments (0–5 cm) for DNA extraction were firstly collected into five 2.5-mL sterile centrifuge tubes (approximately 3g each tubes) using a sterile spatula. Subsequently, the collected sediment samples for DNA extraction were stored on dry ice in the field as well as during transportation. Upon arrival in the laboratory, sediment samples for DNA extraction were immediately stored at −80 ^o^C until further analysis. In addition, pH values of these lakes were measured with a portable pH meter (PT-10, Sartorius, Germany) in the flied. Surface (0–5cm) lake water was first filtered through 0.2 μm Isopore filters (Whatman, UK) and then analyzed for major ions (K^+^, Na^+^, Ca^2+^, Mg^2+^, SO_4_^2−^, Cl^−^, NO_3_^−^ and NH_4_^+^) in the laboratory by using ion chromatography (Dionex DX-600, USA). Salinity was calculated by summarizing the concentrations of major ions. Total organic carbon (TOC) was measured on a multi N/C 2100S analyzer (Analytik Jena, Germany). Before analyzing sediment TOC, samples were firstly acidified with 1 N HCl overnight to remove carbonates, subsequently washed to neutral pH, dried in oven and ground with mortar.

### DNA extraction, polymerase chain reaction and Illumina sequencing

DNA was extracted from 0.5 g sediment samples using the FastDNA SPIN Kit for Soil (MP Biomedical, OH, USA). The extracted DNA was amplified using a set of bar-coded primers 515F and 806R^29^. Triplicate PCR reactions for each sample were conducted and purified using a DNA Gel Extraction Kit (Axygen, CA, USA). The PCR products from each sample were pooled with equimolar concentrations and then sequenced by using an Illumina Miseq platform.

### Sequencing analyses

Demultiplexing and quality filtering of raw sequences were conducted in QIIME^30^. Chimera detection was performed using the UCHIME module of the USEARCH program (usearch_qf, Edgar *et al.* 2011). Operational taxonomic units (OTUs) were defined at 97% sequence similarity by using UCLUST algorithm^31^. OTU representative sequences were picked and their taxonomy were assigned using the ribosome database project (RDP) classifier algorithm^32^. Representative sequences were aligned with the PyNast algorithm against the Greengenes core set (http://greengenes.lbl.gov) and FastTree (http://www.microbesonline.org/fasttree/) was applied to construct a phylogenetic tree. The OTUs comprising only one sequence were removed prior to further analysis to avoid possible biases. Each sample was rarefied to 60000 sequences with 1000 times, and then alpha diversity was calculated at the 97% identity level in QIIME. A variety of alpha diversity indices were calculated including Chao1 (a measure of richness, namely the estimated number of phylotypes), Shannon (includes both richness and evenness), Equitability (i.e. evenness, distribution of phylotypes), and phylogenetic distance whole tree (phylogenetic closeness across the entire tree in a subset of phylotypes). In addition to analysis of different members of a community and their response to environmental factors, we extracted abundant and rare taxa based on OTU relative abundance following previous studies^3^,^33^,^34^. Specifically, the OTUs with relative abundance higher than 1% and lower than 0.01% within one sample were defined as abundant and rare taxa, respectively. Our definition of rare taxa is much more rigorious than previous proposed criteria (<0.1%)^16^, because all of our samples each had more than 60000 reads and the 0.01% threshold can give the almost lowest frequency OTUs that were represented by only several (<10) reads in our samples. In order to assess the difference of microbial community composition (MCC), cluster analysis was performed using the R package “pvclust”^35^ based on Bray-Curtis dissimilarity matrix of the detected OTUs, and Bray-Curtis distance-based principal coordinate analysis (PCoA) was conducted in the R package “ape”^36^. Mantel test was performed to assess the correlations between MCC and environmental parameters by using R package “vegan”^37^.

### Nucleotide sequence accession numbers

The original sequences were deposited at the Sequence Read Archive (NCBI) with accession no. SRS 895733, SRS895834 and SRS895834-895840 under the BioProject: SRP056907.

## Additional Information

**How to cite this article**: Yang, J. *et al.* Salinity shapes microbial diversity and community structure in surface sediments of the Qinghai-Tibetan Lakes. *Sci. Rep.* **6**, 25078; doi: 10.1038/srep25078 (2016).

## Supplementary Material

Supplementary Information

## Figures and Tables

**Table 1:** Geographic and geochemical parameters of the nine studied lakes on the Qinghai-Tibetan Plateau, China.

Sample ID	KLKL	EHL	QHL	TSL	GHL1	GHL2	XDCL	DBXL	CKL
GPS Location (N/E)	37^o^18.7'/	36^o^34.1'/	36^o^38.0'/	37^o^11.6'/	36^o^58.1'/	37^o^7.8'/	37^o^28.8'/	37^o^28.8'/	36^o^45.0'/
	96^o^54.1'	100^o^44.3'	100^o^6.9'	96^o^53.3'	100^o^35.9'	97^o^46.9'	95^o^26.2'	95^o^26.2'	99^o^4.8'
Salinity(g/L)	0.6	0.7	12.9	30.1	30.6	83.6	159.6	307.2	324.8
pH	8.8	9.4	9.1	8.8	8.9	8.4	8.4	7.0	7.8
TOC(%)	5.1	0.3	1.6	3.2	1.2	5.6	0.2	0.4	1.9
**Concentration of major ions (mg L^−1^)**									
K^+^	6.5	9.0	269.2	326.9	613.0	452.9	920.0	2163.0	2089.3
Na^+^	135.2	218.8	3993.0	8087.0	9384.0	26770.0	54691.0	77950.0	107460.3
Ca^2+^	38.5	20.0	17.7	32.4	22.8	392.0	625.0	1711.0	823.2
Mg^2+^	53.6	68.5	824.1	2107.0	1467.0	4097.0	2588.7	24700.0	9740.8
SO_4_^2−^	140.4	117.9	2188.0	6454.0	6058.0	10790.0	28735.2	4462.0	17099.8
Cl^−^	206.2	231.1	5625.7	13129.5	13034.7	41059.4	72063.4	196231.9	187633.2
NO_3_^−^	0.5	0.2	0.4	0.4	0.5	0.6	0.5	1.1	1.4
NH_4_^+^	1.0	1.2	1.0	0.8	0.6	0.5	0.6	0.4	0.4

KLKL: Keluke Lake; EHL: Erhai Lake; QHL:Qinghai Lake; TSL:Tuosu Lake; GHL1:Gahai Lake 1; GHL2: Gahai Lake 2; XCDL: Xiaochaidan Lake; DBXL: Dabuxun Lake; CKL: Chaka Lake.
