# Supplementary material for: Salinity shapes microbial diversity and community structure in surface sediments of the Qinghai-Tibetan Lakes
Source: Sci Rep. 2016 Apr 26;6:25078. doi: 10.1038/srep25078 (PMC4844989; doi:10.1038/srep25078)

**Salinity shapes microbial diversity and community structure in surface sediments of the Qinghai-Tibetan Lakes**

*Running title: Salinity shapes Microbial Community in Tibetan lakes*

Jian Yang1^, Li'an Ma2^, Hongchen Jiang^1*^, Geng Wu^1^ and Hailiang Dong^1,3*^,

^1^*State Key Laboratory of Biogeology and Environmental Geology, China University of Geosciences, Wuhan, 430074, China*

^2^*State Key Laboratory of Biogeology and Environmental Geology, China University of Geosciences, Beijing, 100083, China*

^3^*Department of Geology and Environmental Earth Science, Miami University, Oxford, OH 45056, USA*

^Contribute equally

*Corresponding authors:

Hongchen Jiang: [jiangh@cug.edu.cn](mailto:jiangh@cug.edu.cn); Tel: 86-27-67883452

Hailiang Dong: [dongh@cug.edu.cn](mailto:dongh@cug.edu.cn) or [dongh@miamioh.edu](mailto:dongh@miamioh.edu)

Revised for Scientific Reports

March 31, 2016

Table S1 Alpha-diversity of the sediments in the studied Qinghai-Tibetan lakes

| **Sample** | **Total reads** | **Observed OTUs** | **Simpson** | **Shannon_Wiener** | **PD_whole_tree** | **Equitability** | **Chao1** |
| --- | --- | --- | --- | --- | --- | --- | --- |
| **KLKL** | 61798 | 3088.5 | 1.0 | 8.9 | 92.7 | 0.8 | 3288.7 |
| **EHL** | 71592 | 2978.8 | 1.0 | 9.1 | 93.3 | 0.8 | 3356.4 |
| **QHL** | 87290 | 2316.7 | 1.0 | 7.4 | 72.7 | 0.7 | 2851.4 |
| **TSL** | 62097 | 2171.4 | 1.0 | 8.4 | 78.4 | 0.8 | 2323.8 |
| **GHL1** | 71621 | 1923.4 | 1.0 | 8.2 | 65.7 | 0.8 | 2233.8 |
| **GHL2** | 70686 | 2573.1 | 0.9 | 7.4 | 85.3 | 0.6 | 2946.9 |
| **XCDL** | 69647 | 1860.0 | 1.0 | 7.4 | 67.1 | 0.7 | 2142.5 |
| **DBXL** | 100419 | 581.5 | 0.9 | 4.7 | 11.2 | 0.5 | 695.0 |
| **CKL** | 107066 | 768.0 | 0.9 | 5.6 | 17.9 | 0.6 | 930.2 |

**Table S2** Abundance estimates of the abundant and rare OTUs in the studied lake surface sediment samples in this study

| Sample | Abundant OTUs  (Percentage of  abundant OTUs/ total OTUs  in each sample) | Abundant OTU  relative abundance (%) | Rare OTUs  (Percentage of  rare OTUs/ total OTUs  in each sample) | Rare OTU  relative abundance (%) |
| --- | --- | --- | --- | --- |
| KLKL | 8 (0.3%) | 24.4 | 1997 (64.4%) | 8.7 |
| EHL | 11 (0.4%) | 20.4 | 1989 (64.5%) | 7.9 |
| QHL | 16 (0.6%) | 49.4 | 1928 (75.3%) | 6.1 |
| TSL | 15 (0.7%) | 32.0 | 1306 (59.8%) | 5.5 |
| GHL1 | 17 (0.9%) | 32.7 | 1268 (63.5%) | 4.7 |
| GHL2 | 9 (0.3%) | 41.4 | 1861 (69.8%) | 7.2 |
| XCDL | 17 (0.9%) | 48.1 | 1274 (66.2%) | 4.5 |
| DBXL | 19 (2.9%) | 78.8 | 426 (78.8%) | 1.5 |
| CKL | 19 (2.2%) | 67.5 | 601 (68.9%) | 1.9 |

**Table S3** Relative abundance of abundant OTUs within different phyla across the studied samples in this study

| **Phylum** | **KLKL** | **EHL** | **QHL** | **TSL** | **GHL1** | **GHL2** | **XCDL** | **DBXL** | **CKL** |
| --- | --- | --- | --- | --- | --- | --- | --- | --- | --- |
| ***Proteobacteria*** | 22.91 | 18.16 | 16.69 | 18.22 | 25.81 | 38.54 | 33.09 | 60.87 | 44.70 |
| ***Bacteroidetes*** | 0.00 | 2.20 | 8.33 | 3.64 | 1.05 | 1.25 | 6.17 | 8.29 | 3.82 |
| ***Thermi*** | 0.00 | 0.00 | 5.86 | 3.55 | 3.68 | 0.00 | 4.16 | 0.00 | 0.00 |
| ***Cyanobacteria*** | 0.00 | 0.00 | 9.27 | 0.00 | 0.00 | 0.00 | 2.68 | 0.00 | 4.56 |
| ***Firmicutes*** | 0.00 | 0.00 | 0.00 | 0.00 | 0.00 | 0.00 | 0.00 | 2.38 | 10.63 |
| ***Verrucomicrobia*** | 0.00 | 0.00 | 9.28 | 0.00 | 0.00 | 0.00 | 0.00 | 0.00 | 0.00 |
| ***Actinobacteria*** | 0.00 | 0.00 | 0.00 | 3.30 | 0.00 | 0.00 | 1.98 | 1.01 | 0.00 |
| ***Chloroflexi*** | 1.45 | 0.00 | 0.00 | 0.00 | 1.12 | 0.00 | 0.00 | 0.00 | 1.47 |
| ***Gemmatimonadetes*** | 0.00 | 0.00 | 0.00 | 2.06 | 1.04 | 0.00 | 0.00 | 0.00 | 0.00 |
| ***Acidobacteria*** | 0.00 | 0.00 | 0.00 | 1.23 | 0.00 | 1.57 | 0.00 | 0.00 | 0.00 |
| **OP8** | 0.00 | 0.00 | 0.00 | 0.00 | 0.00 | 0.00 | 0.00 | 0.00 | 2.30 |

**Table S4** Relative abundance of rare OTUs within different phyla across the studied samples in this study

| **Phylum** | **KLKL** | **EHL** | **QHL** | **TSL** | **GHL1** | **GHL2** | **XCDL** | **DBXL** | **CKL** |
| --- | --- | --- | --- | --- | --- | --- | --- | --- | --- |
| ***Proteobacteria*** | 3.50 | 3.23 | 2.82 | 2.11 | 2.43 | 3.09 | 1.82 | 0.84 | 1.15 |
| ***Bacteroidetes*** | 0.92 | 0.83 | 0.75 | 0.63 | 0.45 | 0.78 | 0.55 | 0.09 | 0.13 |
| ***Actinobacteria*** | 0.44 | 0.67 | 0.52 | 0.37 | 0.32 | 0.42 | 0.28 | 0.17 | 0.11 |
| ***Firmicutes*** | 0.42 | 0.38 | 0.36 | 0.25 | 0.22 | 0.37 | 0.24 | 0.31 | 0.37 |
| ***Chloroflexi*** | 0.66 | 0.47 | 0.26 | 0.24 | 0.13 | 0.51 | 0.19 | 0.00 | 0.00 |
| ***Planctomycetes*** | 0.43 | 0.36 | 0.27 | 0.38 | 0.21 | 0.27 | 0.22 | 0.00 | 0.00 |
| ***Verrucomicrobia*** | 0.48 | 0.42 | 0.26 | 0.20 | 0.23 | 0.19 | 0.15 | 0.00 | 0.01 |
| ***Acidobacteria*** | 0.33 | 0.38 | 0.22 | 0.09 | 0.11 | 0.34 | 0.08 | 0.00 | 0.01 |
| ***Cyanobacteria*** | 0.29 | 0.20 | 0.20 | 0.19 | 0.20 | 0.05 | 0.13 | 0.01 | 0.01 |
| ***Euryarchaeota*** | 0.22 | 0.14 | 0.07 | 0.30 | 0.03 | 0.09 | 0.22 | 0.01 | 0.08 |
| **Others** | 1.02 | 0.85 | 0.41 | 0.79 | 0.43 | 1.11 | 0.58 | 0.02 | 0.04 |

**Table S5** Relative abundance (%) of top 10 classes in the sediments of the studied Qinghai-Tibetan lakes

| **Top 10 classes** | **KLKL** | **EHL** | **QHL** | **TSL** | **GHL1** | **GHL2** | **XCDL** | **DBXL** | **CKL** |
| --- | --- | --- | --- | --- | --- | --- | --- | --- | --- |
| ***Gammaproteobacteria*** | 12.6 | 5.8 | 14.1 | 15.6 | 9.0 | 4.7 | 10.8 | 67.4 | 49.6 |
| ***Betaproteobacteria*** | 28.7 | 22.0 | 8.4 | 1.6 | 22.1 | 41.9 | 0.3 | 2.6 | 8.5 |
| ***Deltaproteobacteria*** | 10.2 | 13.6 | 9.5 | 16.1 | 12.6 | 16.1 | 31.0 | 0.0 | 0.0 |
| ***Alphaproteobacteria*** | 2.5 | 4.5 | 8.8 | 11.5 | 10.4 | 1.7 | 12.7 | 3.9 | 5.5 |
| ***Bacteroidia*** | 6.5 | 10.1 | 4.7 | 4.8 | 5.2 | 6.5 | 11.0 | 0.7 | 0.0 |
| ***Flavobacteriia*** | 0.9 | 0.7 | 6.6 | 1.3 | 5.1 | 1.1 | 1.2 | 9.9 | 5.0 |
| ***Anaerolineae*** | 8.9 | 6.8 | 0.5 | 1.2 | 2.6 | 3.7 | 1.7 | 0.0 | 0.0 |
| ***Clostridia*** | 1.1 | 1.2 | 1.1 | 0.8 | 3.1 | 0.8 | 3.0 | 2.4 | 11.3 |
| ***Verrucomicrobiae*** | 3.4 | 2.5 | 11.5 | 1.0 | 3.4 | 0.1 | 0.3 | 0.0 | 0.0 |
| ***Deinococci*** | 0.0 | 1.4 | 5.9 | 4.4 | 3.8 | 0.1 | 4.5 | 0.0 | 0.2 |

**Table S6** Mantel test showing the correlation between MCC similarity and environment parameters of the studied lakes in this study

|  | All OTUs | Abundant OTUs | Rare OTUs |
| --- | --- | --- | --- |
| Geographic distance | -0.056 | -0.033 | -0.050 |
| Salinity | 0.631** | 0.427* | 0.783** |
| pH | 0.402* | 0.201 | 0.574* |
| TOC | -0.010 | 0.027 | -0.118 |

The Pearson’s coefficients were calculated and their significances were tested based on 999 permutations.

* *P*< 0.05 and ***P*< 0.01.

**Caption:**

Fig. S1 Bray-Curtis dissimilarity-based cluster analysis (left) of MCC in the studied samples and schematic figures (right) showing the frequencies of OTUs affiliated with major phyla in this study.

Fig. 2 Cluster analyses and principal coordinates analyses of abundant and rare MCC among the studied samples based on Bray-Curtis dissimilarity.

Fig. S1


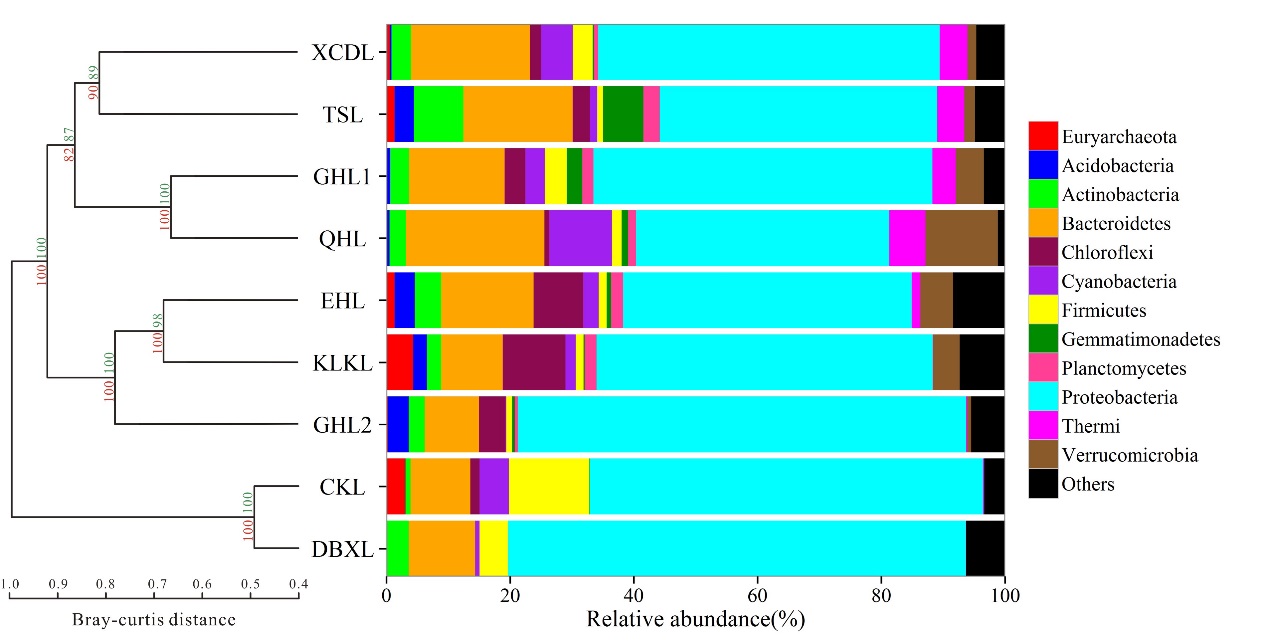


Fig. S2


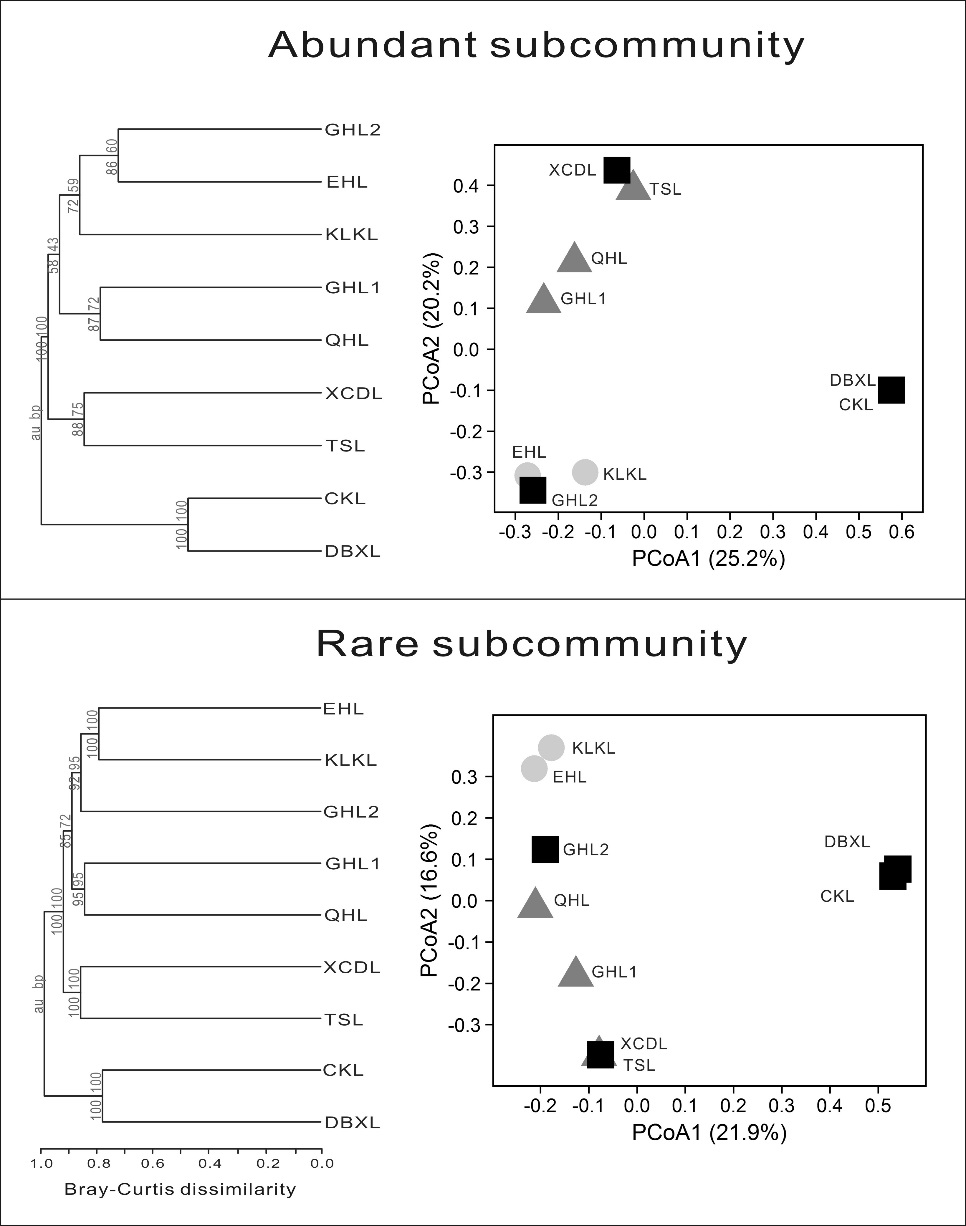

Supplement: Supplementary Information [file srep25078-s1.doc]
